# China’s 1-3-7 surveillance and response strategy for malaria elimination: Is case reporting, investigation and foci response happening according to plan?

**DOI:** 10.1186/s40249-015-0089-2

**Published:** 2015-12-10

**Authors:** Shui-Sen Zhou, Shao-Sen Zhang, Li Zhang, Aafje E. C. Rietveld, Andrew R. Ramsay, Rony Zachariah, Karen Bissell, Rafael Van den Bergh, Zhi-Gui Xia, Xiao-Nong Zhou, Richard E. Cibulskis

**Affiliations:** National Institute of Parasitic Diseases, Chinese Center for Disease Control and Prevention; Key Laboratory of Parasite and Vector Biology, MOH; WHO Collaborating Centre for Tropic Diseases, National Center for International Research on Tropical Diseases, 207 Rui Jin Er Road, Shanghai,, 200025 People’s Republic of China; Global Malaria Programme, World Health Organization, 20 Avenue Appia, CH-1211, Geneva, 27 Switzerland; Special Programme for Research and Training in Tropical Diseases (TDR), 20 Avenue Appia, CH-1211, Geneva, 27 Switzerland; Médecins Sans Frontieres, Brussels Operational Centre, Luxembourg, Luxembourg; International Union Against Tuberculosis and Lung Disease, Paris, France

**Keywords:** 1-3-7 strategy, Malaria elimination, Surveillance and response

## Abstract

**Background:**

The China’s 1-3-7 strategy was initiated and extensively adopted in different types of counties (geographic regions) for reporting of malaria cases within 1 day, their confirmation and investigation within 3 days, and the appropriate public health response to prevent further transmission within 7 days. Assessing the level of compliance to the 1-3-7 strategy at the county level is a first step towards determining whether the surveillance and response strategy is happening according to plan. This study assessed if the time-bound targets of the 1-3-7 strategy were being sustained over time. Such information would be useful to improve implementation of the 1-3-7 strategy in China.

**Methods:**

This cross-sectional study involved country-wide programmatic data for the period January 1st 2013 to June 30th 2014. Data variables were extracted from the national malaria information system and included socio-demographic information, type of county, date of diagnosis, date of reporting, date of case investigation, case classification (indigenous, or imported, or unknown), focus investigation, date of reactive case detection (RACD), and date of indoor residual spraying (IRS). Summary statistics and proportions were used and comparisons between groups were assessed using the chi-square test. Level of significance was set at a *P*-value ≤ 0.05.

**Results:**

Of a total of 5,688 malaria cases from 731 counties, there were 55 (1 %) indigenous cases (only in Type 1 and Type 2 counties) and 5,633 (99 %) imported cases from all types of counties. There was no delay in reporting malaria cases by type of county. In terms of case investigation, 97.5 % cases were investigated within 3 days with the proportion of delays (1.5 %) in type 2 counties, being significantly lower than type 1 counties (4.1 %). Regarding active foci, 96.4 % were treated by RACD and/or IRS.

**Conclusions:**

The performance of 1-3-7 strategy was encouraging but identified some challenges that if addressed can further improve implementation.

**Electronic supplementary material:**

The online version of this article (doi:10.1186/s40249-015-0089-2) contains supplementary material, which is available to authorized users.

## Multilingual abstracts

Please see Additional file 1 for translations of the abstract into the six official working languages of the United Nations.

## Background

Malaria elimination depends on surveillance systems that can rapidly and efficiently detect, treat and respond to individual cases in the population. Success in doing so will determine whether or not malaria elimination can be achieved and sustained. The most widely adopted approach to surveillance and response is a strategy termed reactive case detection (RACD), whereby household members, neighbor’s, and other contacts of passively detected malaria cases are screened for infection and are treated with effective antimalarial drugs [1–3]. Thirteen of 14 countries in the Asia Pacific region and several countries in Africa [4–7] including Swaziland, South Africa, Zambia, Namibia and Senegal, and countries in the malaria elimination phase employ some form of RACD.

China launched malaria elimination program in 2010 with the goal of achieving country-wide elimination by 2020. The elimination goal was formulated in accordance with a four-catalogue classification of transmission settings and on the basis of malaria transmission risk and incidence at county level. Counties were categorized into three types. Type 1 having local transmission and incidence ≥1/10,000 in the past 3 years, Type 2 having local transmission and incidence <1/10,000 in the past 3 years, Type 3 no indigenous cases reported in the past years but still with risk of transmission, and Type 4 which are malaria free [8]. The total number of malaria cases including indigenous and imported cases in the country has reduced dramatically from over 26,000 in 2008 to 2,716 in 2012. Of the latter, only 243 cases (accounting for 8.9 %) were designated as being indigenously acquired [9]. This significant reduction of indigenous malaria incidence has been attributed to the program using an adapted form of RACD in surveillance and response. This adapted form is called the “1-3-7” strategy and has time-bound targets for case reporting, investigation, and foci response activities. The “1-3-7” refers respectively to reporting of malaria cases within one day, their confirmation and investigation within 3 days, and the appropriate public health response to prevent further transmission within 7 days.

Before 2010 and in accordance with the national law, as a notifiable disease (Category B), all malaria cases including clinical and laboratory confirmed cases must be registered and reported by health facilities. This is done through the Chinese Information System for Disease Control and Prevention (CISDCP), an internet-based reporting system. With the launch of the NMEP, the Information Management System Specific to Malaria Elimination (IMSME) and a cellphone-based SMS alert system were incorporated with CISDCP, all information including the laboratory test and classification based on the epidemiological individual survey were required to be reported in the system in a timely manner. Once a malaria case is reported to the system, CDC’s staffs at the county level were deployed to carry out an individual investigation in the field to identify the source of infection and whether this was an indigenous or imported case. For this, a blood smear is taken from the case and sent to the national CDC and the provincial CDCs for microscopic verification. A blood-spot filter paper sample is also delivered to the reference laboratories in provincial CDCs and national CDC for molecular verification using polymerase chain reaction (PCR).

Case classification is based on guidelines of the World Health Organization (WHO) [10] and includes an indigenous case, imported case and unknown. An indigenous case is defined as a case contracted indigenously (i.e. within national boundaries) while an imported case is one where the origin can be traced to a known malaria endemic area outside the national borders to where the case has travelled within one month. This information is elicited on the basis of the travel history of the patient. When information is not available on the origin of the case, it is classified as “unknown”. The results have to be entered into the IMSME. The details of this system have been described in a previous publication [11].

**Fig. 1:**
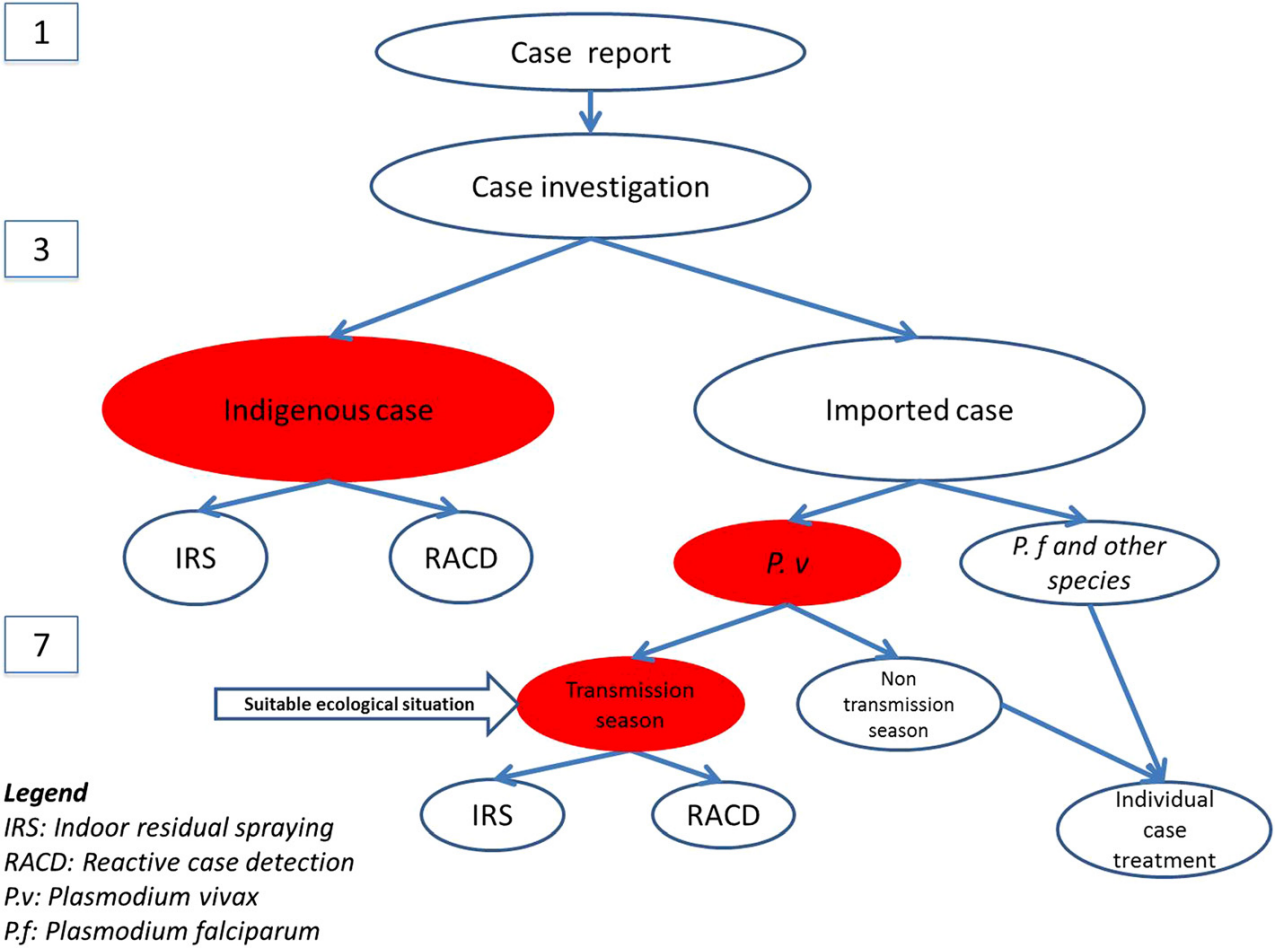
The flowchart of 1-3-7 strategy implementation (**IRS:** indoor residual spraying. **RACD:** reactive case detection. **P.v:** *Plasmodium vivax*. **P.f:** *Plasmodium falciparum*. **1-3-7 strategy:** A diagnosed malaria case will be reported within 1 day, and a case investigation will be conducted within 3 days to identify whether the case is indigenous or imported case for further response. The focus caused by the indigenous case is considered as an active focus with response activities such as IRS and RACD that will be done within 7 days. **Classification of focus:** the imported *P. vivax* cases occurred in the transmission season will be responded as active focus due to the possibility of local transmission, while imported *P. falciparum* cases and other species as well as *P. vivax* cases occurred in non-transmission season would be treated as inactive focus with only individual case treatment due to no transmission conditions)

Normally all imported and indigenous cases need to be investigated and focus investigation should be done in 7 days where there is a transmission risk. This is not required for imported *Plasmodium falciparum* cases in China as due to the lack of efficient vectors to sustain *P. falciparum* and its further transmission (the predominant imported malaria parasite) [12]*.* However if the imported case is *P. vivax* and the area has malaria transmission risk based on favorable entomological and ecologic conditions (transmission season), focus investigation is supposed to be done (Fig. 1).

Case reporting and case investigation as well as the foci investigation have to be conducted in all counties, whereas RACD and indoor residual spraying (IRS) are only needed for active foci. RACD will be done in the household where the case was identified and neighboring households within a 300 m radius if the focus is considered large (an entire village). In case the focus area is small, then all households will be screened. IRS would be conducted only in index household and neighboring households to ensure compliance. Table 1 shows the components of the 1-3-7 strategy designed to guide and monitor malaria surveillance and response in China [11].

**Table 1:** The 1-3-7 strategy designed to guide malaria surveillance and response in China

Strategy	Time required	Guidance in surveillance and response
*Case reporting within 1 day*	Day 1	Any confirmed and suspected malaria cases by law must be reported to the Chinese Center for Disease Control and Prevention (CDC) through the web-based health information system within 24 h of diagnosis by the local health-care provider.
*Case investigation within 3 days*	Day 3	All malaria cases should be confirmed and visited by CDC’s staff at the county-level China Center for Disease Control (CDC), where the case is reported within three days, to determine where the case originated (indigenous or imported).
*Focus investigation and action within 7 days*	Day 7	The focus investigation should be conducted as soon as possible. If local transmission* is possible or confirmed, targeted action to seek out other infections and reduce the chance of onward transmission is completed within seven days by the China CDC’s staff of the county where the patient resides and/or works. The scope of investigation is the case house and neighboring households.
		*: Only these are considered active foci.

This strategy was developed by the national malaria elimination program since 2010 when the program launched. It was extensively rolled out nationally in early 2012.

Implementation of the 1-3-7 strategy may be facing operational challenges in various counties in different geographic zones across China. For example, there might be delays in laboratory confirmation and household investigation of cases and their household members due to short-comings in the web-based health information system, e.g. breakdown of cellphones or network coverage problems. Other possible reasons for delays may include late field visits by CDC’s staff at the county level caused by logistic hurdles. There may also be differences in compliance between counties due to variations in geographic access or due to the catalogue of county with different transmission status.

Assessing the level of compliance to the 1-3-7 strategy at the county level is the first step towards determining whether the surveillance and response strategy is happening according to plan. An earlier study [11] provided an overview of the overall performance of the 1-3-7 strategy in China following the first year of implementation. There was however no systematic assessment of whether there were variations in implementation between different counties classified as Type 1 to 4 on the basis of malaria transmission risk and incidence. Moreover, the previous study could not fully present the performance because of incompletion of data as the early development of the strategy and the reporting system. For example, the proportion of foci response within 7 days was only about 50 % which deflected the real situation. Continued country-wide assessments are needed to verify if the time-bound targets of the 1-3-7 are being sustained over time. Such information would be useful to improve implementation of the 1-3-7 strategy in China.

We therefore conducted a country-wide study to determine whether China’s 1-3-7 malaria surveillance strategy was being implemented according to the planned schedule of reporting, case investigation and focus response and if there were any differences between types of counties.

## Methods

### Country-wide study

A cross-sectional study involving country-wide programmatic data extracted from the national malaria information system in China was performed.

China has a population of 1.37 billion, which is distributed unevenly, with more in the east (>300 persons per square kilometer) and fewer in the west (about 40 persons per square kilometer). The average population density is 119 per square kilometer, with an average household size of 3.7 persons. There are 2,858 counties in mainland China, which are grouped into 31 administrative areas (including provinces, municipalities and autonomous regions).

### Local study setting

Yunnan Province is located in the Greater Mekong Sub-region, sharing the long border with Myanmar, Lao PDR and Viet Nam which have a high malaria burden. The number of malaria cases reported in Yunnan contributed to most cases reported in China. Because of the high risk of malaria transmission and developing traffic network, the performances of malaria elimination as well as the implementation of 1-3-7 strategy in Yunnan are considered to be important. Therefore, Yunnan Province was selected as a study site to evaluate the performance of the 1-3-7 strategy in the local settings, in addition to the country-wide study.

### Implementations of NMEP in different types of counties

According to the National Malaria Elimination Action Plan 2010–2020 [8], different strategic priorities are implemented in different types of counties. The Type 1 counties should strengthen case management and vector control measures to reduce the incidence of malaria. The Type 2 countries should eliminate the infectious source of malaria to interrupt local malaria transmission. The Type 3 counties should enhance the monitoring and surveillance on the imported cases to prevent the secondary transmission. The Type 4 counties should sensitively detect and promptly respond to the imported cases.

### Data extraction and validation

All patients diagnosed as malaria cases from 1 January 2013 to 30 June 2014 were included in the study. Data variables were extracted from the NMIS, which is accessible at county, provincial and central levels. Data variables included basic socio-demographic information about patients, type of county, date of diagnosis, date of reporting, date of case investigation, case classification including indigenous, imported and unknown cases, focus investigation, date of RACD, date of IRS.

Data on source of infection including indigenous, imported or unknown cases is entered in the NMIS by staff in the national CDC while PCR data is entered at province level.

### Data analysis

Summary statistics and proportions were used and comparisons were made between different types of counties using chi-square test. Level of significance was set at *P*-value ≤ 0.05.

The causes of differences in different types of counties were analyzed using Yunnan provincial data, while the chi-square test was used to assess statistically differences between groups.

### Ethics approval

Ethics approval was received from the National Institute of Parasitic Diseases, China Center for Disease Control and Prevention, and the local Centers for Disease Control and Prevention. Approval was also received from the Ethics Advisory Group of the International Union against Tuberculosis and Lung Disease, Paris, France.

## Results

### Malaria cases reported in China stratified by type of county

**Fig. 2:**
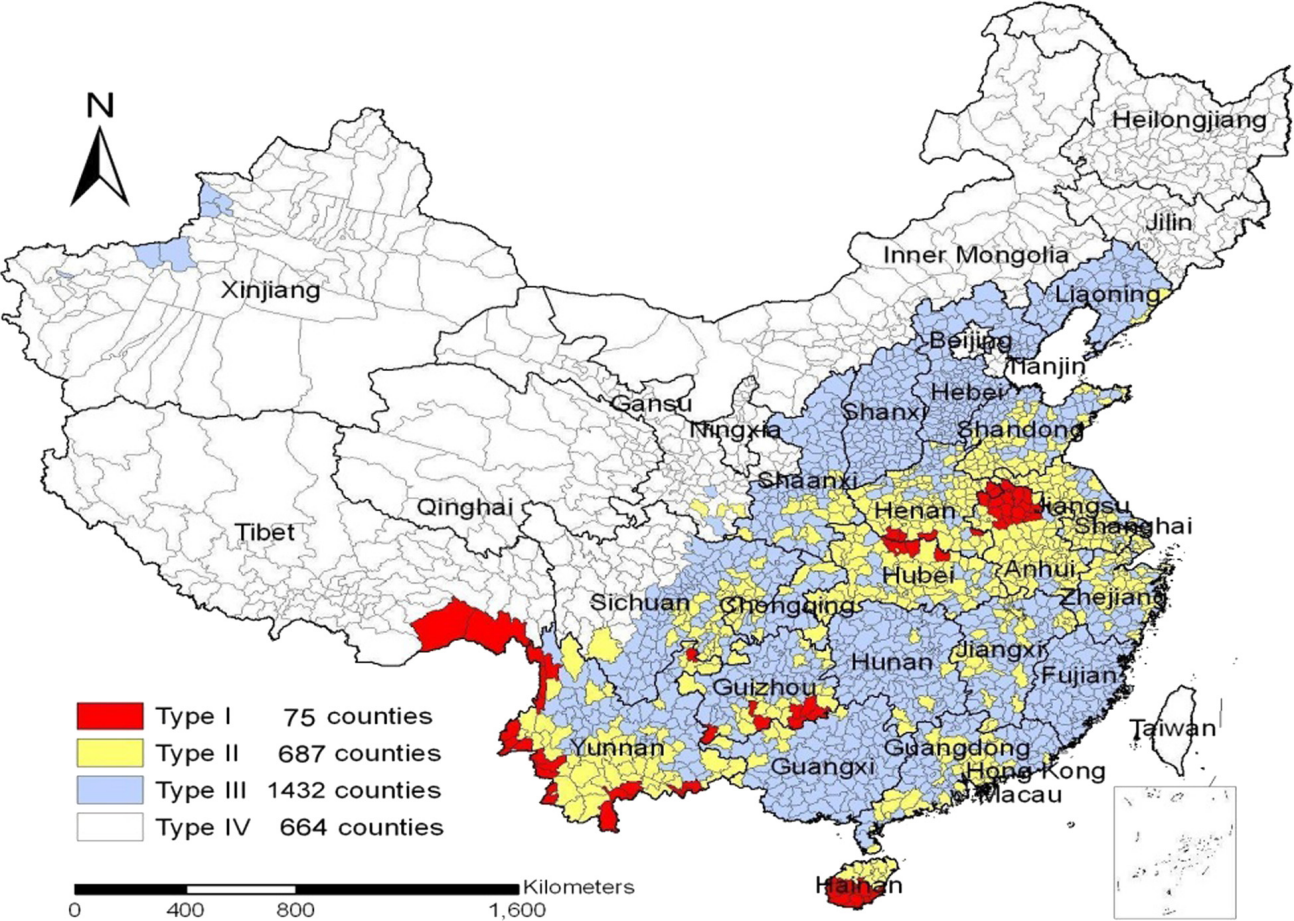
Stratification of malaria counties in relation to malaria transmission risk and incidence in China (2010)

The number of counties stratified by type according to malaria transmission risk is summarized in Fig. 2. Out of a total of 2858 counties, 75 were Type 1 counties, 687 were Type 2, 1,432 were Type 3, and 664 were Type 4. A total of 731(about 26 %) counties reported a total of 5,688 malaria cases, 99 % being imported cases and 1 % indigenous (Table 2).

**Table 2:** The numbers of various malaria cases in different types^a^ of county in China, January 2013-June 2014

Type of county^a^	Counties	Cases			
	No (%)	Indigenous^b^ No (%)	Imported^c^ No (%)	Unknown No (%)	Total
Type 1	38 (5.2)	52 (10.6)	440 (89.4)	0	492
Type 2	305 (41.7)	3 (0.1)	2027 (99.9)	0	2030
Type 3	349 (47.8)	0	2939 (100)	0	2939
Type 4	39 (5.3)	0	227 (100)	0	227
Total	731 (100)	55 (1)	5633 (99)	0	5688

^a^Type of county:

Type 1: having local transmission and incidence >1/10,000 in the past 3 years

Type 2: having local transmission and incidence <1/10,000 in the past 3 years

Type 3: no local cases reported in the past years but still with risk of transmission

Type 4: malaria free

^b^An indigenous case is defined as a case contracted indigenously (i.e. within national boundaries)

^c^The origin can be traced to a known malaria endemic area outside the national borders to where the case has travelled within one month

### Case reporting within one day

All cases were reported within 1 day of diagnosis irrespective of the type of county.

### Case investigation within three days

All reported malaria cases were individually investigated using a questionnaire by CDC’s staff at the county level, with 97 % done within 3 days (Table 3). In the remainder (3 %) there were delays of more than 3 days. Delays ranged from 1.5 to 4 % of cases and were seen in all counties. The number of cases that faced delays in Type 2 counties was significantly lower than in that of Type 1 and Type 3. However, on difference occurred in the median delay in days among each type (Table 3).

**Table 3:** The number of malaria cases investigated in different types of county in China, January 2013-June 2014

Type of county	Total cases	Case investigation		
		≤3 days	>3 days	
		No (%)	No (%)	Days of delay (Median)
Type 1	492	472 (96)	20 (4)	5
Type 2	2030	1999 (98)	31 (2)	6
Type 3	2939	2851 (97)	88 (3)	6
Type 4	227	222 (98)	5 (2)	6
Total	5688	5544 (97)	144 (3)	5

*χ* ^2^ (Type 1 vs Type 2) = 12.87, *P* = 0.0003^*^

*χ* ^2^ (Type 1 vs Type 3) = 1.59, *P* = 0.208

*χ* ^2^ (Type 1 vs Type 4) = 1.60, *P* = 0.205

*χ* ^2^ (Type 2 vs Type 3) = 11.06, *P* = 0.0009^*^

Fisher (Type 2 vs Type 4): *P* = 0.401

Fisher (among groups): *P* = 0.0013

*P<0.05

**Fig. 3:**
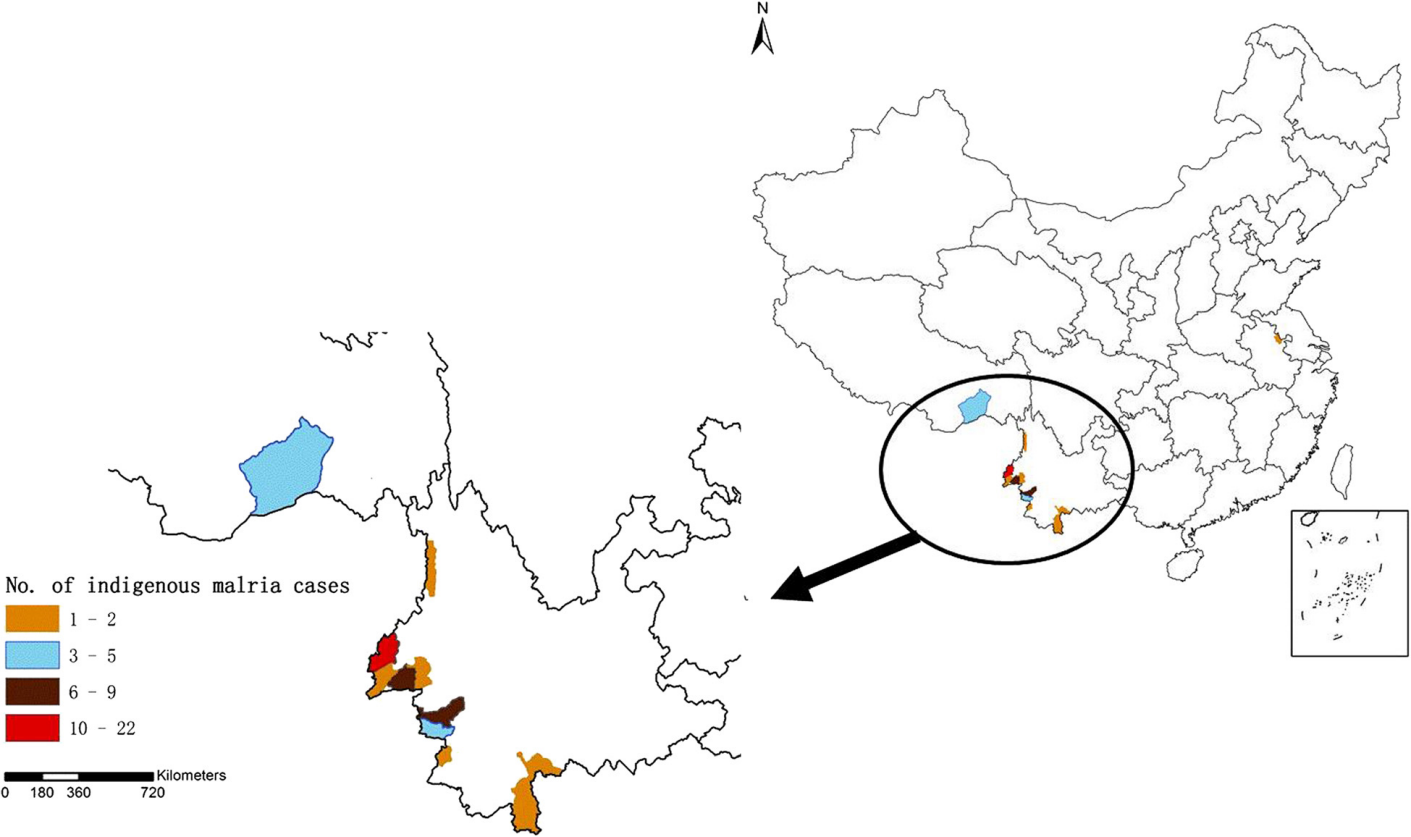
Distribution of indigenous malaria cases China, January 2013-June 2014

The 55 indigenous cases were limited to 13 counties involving three provinces/autonomous regions. Among them, 49(accounting for 89 %) cases were found from 11 border counties in Yunnan Province, four cases from one county in Tibet and two cases from one county in Anhui Province (Fig. 3).

### Foci response within seven days

The active foci caused by 55 indigenous cases were identified and these were all located in Type 1 and Type 2 counties. Among them, 53 received responses with RACD and IRS within 7 days and in two foci both RACD and IRS were missed (Table 4). Consolidation of data on focus investigation and response for imported cases was met with challenges because of the lack of uniform implementation guidelines among all countries in the country.

**Table 4:** The number of focus investigation and response in different types of county in China, January 2013-June 2014

Type of county	Total	No response^b^	Focus response^a^	
			≤7 days	>7 days
Type 1	52	2	50	0
Type 2	3	0	3	0
Type 3	0	0	0	0
Type 4	0	0	0	0
Total	55	2	53	0

^a^Focus response would be conducted when an active focus was identified i.e. a village or a community found to have an indigenous case

^b^No response refers to neither RACD nor IRS done in an active focus

### The postponement of case investigation in Yunnan local settings

There were 576 malaria cases reported in Yunnan Province in the study period. Among them, only 37 (6 %) cases faced delayed investigation. The proportion of delayed investigation among different types of counties was not significantly different (Table 5).

**Table 5:** The postponement of case investigation in different types of county in Yunnan Province, January 2013-June 2014

Type of county	Total cases	Case investigation		
		≤3 days	>3 days	
		No (%)	No (%)	Days of delay (Median)
Type 1	299	280 (94)	19 (6)	5
Type 2	228	212 (93)	16 (7)	5
Type 3	49	47 (96)	2 (4)	6
Total	576	539 (94)	37 (6)	5

*χ* ^2^ = 0.583, *P* = 0.747

## Discussion

It is the first time to assess the country-wide performance of the 1-3-7 strategy for malaria elimination in China which shows encouraging results. All malaria cases were reported within the first 24 h, 98 % of case investigations were done within 3 days, 96 % of foci investigations were done in 7 days, and 96 % of active foci of indigenous cases were offered treatment/RACD and IRS according to the planned schedule. There were no significant differences between Type 1, 2 and 3 counties in terms of case investigation as well as foci investigation and response. Moreover, even in the rural areas with highly risk areas for malaria transmission, such as Yunnan Province, the similar performance as the whole country was observed.

Findings from this study showed that the ambitious time-bound targets for reporting, case investigation and foci response can be achieved in a large and diverse country like China. This provides promising hope towards achievement of the goal of malaria elimination by 2020 and adds political impetus to other countries in Asia and Africa regions that are either considering or have already adopted similar approaches to malaria elimination [4–7]. Especially this 1-3-7 strategy is able to be applied in GMS countries where has similar malaria transmission risk and nature environment as that of Yunnan Province.

In fact, 1-3-7 strategy could not to be adapted directly to the different local settings totally [13–19]. For example, it would be not possible to report all malaria cases within 24 h if without the reliable web-based information system for disease control and prevention available in China, thanks to the wide cellphone provider network and cellphone-based SMS alert system that promptly informs the local CDC at the county level. While in countries where this network is less reliable or inexistent the time frame of 24 h used for reporting cases may need adjustment [20–26]. For example, if health facilities do not have a phone or VHF radio then the time needed for information transfer, may be increased.

An operational consideration is the presence of two parallel case reporting systems related to malaria in China, including NIDRIS and IMSME. The NIDRIS covers all hospitals and CDCs at various levels and is responsible for all notifiable infectious diseases including basic information on malaria. On the other hand, the IMSME is specific to malaria elimination which including records of all elimination performance activities. IMSME was recently developed for CDCs to verify and confirm reports from the NIDRIS as a complement component. Obviously, suspected and clinical malaria cases are also reported by hospitals covered by the NIDRIS. This constitutes duplicate work as both systems are currently being cross-checked and validated by county CDC staff. It thus adds to unnecessary and time-consuming workload in some areas. There is a need to rationalize this reporting, while at the same time ensuring validity.

Case investigation, including case classification within 3 days, is a vital step in the 1-3-7 strategy and it is especially important to determinate the infectious sources which provides information for further investigation and intervention. Although we achieved the 3-day target in 98 % of cases, understandably this step is most prone to logistic or other difficulties. Rural areas risk facing delays as the various activities [27–33]; sample collection (blood smears and filter paper); their expedition by hospital staff to local CDC’s staff; and PCR confirmatory testing at centralized laboratories followed by household investigation may need more than 3 days. The need for additional transport and other resources may need to be considered and merits investigation.

Ideally, case investigation at the household level should be conducted by local CDCs’ staffs, but this was also done by busy hospital doctors in some provinces. This may have contributed influenced quality of data. According to the results in Table 5, better coordination between CDC and hospitals is needed to ensure a concerted response which should primarily fall under the mandate of the CDC.

Although we achieved a 96 % success in terms of foci response for indigenous cases within 7 days, one of the challenges we noted was the difficulty in consolidating data on focus investigation and response for the imported cases. This can be attributed to the lack of clear implementation guidelines for county staff on whether or not focus investigation should be conducted on the basis of local entomological and ecological factors that favor transmission. In light of the aforementioned findings, we have preferred not to include this data in our analysis. As the numbers of imported *P. vivax* cases in China are few [16, 34], this short-coming is unlikely to have significant public health impact, it merits focused attention. Operational guidelines are needed to improve guidance on implementation of focus investigation and response [33, 35–37].

The main strength of this study is the inclusion of country-wide data collected under operational conditions and stratified by types of county. Meanwhile, Yunnan Province was exemplified as a case-study because of its special situation and top priority in the NMEP: Many factors would be challenges in the performance of the 1-3-7 strategy, such as indigenous cases, falciparum malaria transmission, borderlines, migrants, minorities and poor transportation. Whether the implementation is feasible in Yunnan Province would be crucial to evaluate the full performance of the strategy in the whole country. The findings are thus likely to reflect the real situation in both particular settings and the context of the whole country.

Study limitations are that we did not know the exact reasons for delays both in case investigation and foci response. Although these were relatively minimal, they still merit specific investigation and correction. Furthermore, we classified 55 cases as being indigenous ones based solely on travel history. This may lead to some degree of misclassification particularly of *P. vivax* cases which might be due to relapsed or recrudescent cases. Genotyping that can distinguish the geographical origin of infections may improve any classification errors.

## Conclusion

In conclusion, the 1-3-7 strategy for malaria elimination being implemented in the NMEP of China is well performed as expected in spite of some challenges in local settings. This is an “eye opener” and may imply that similar standardized and time-bound strategies maybe used to implement and monitor other disease elimination programs at the global level.

## Open access statement

In accordance with WHO’s open-access publication policy for all work funded by WHO or authored/co-authored by WHO staff members, the WHO retains the copyright of this publication through a Creative Commons Attribution IGO licence (http://creativecommons.org/licenses/by/3.0/igo/legalcode) which permits unrestricted use, distribution and reproduction in any medium provided the original work is properly cited.

## Additional file

Additional file 1:
**Multilingual abstracts in the six official working languages of the United Nations.** (PDF 278 kb)
