# Supplementary material for: China’s 1-3-7 surveillance and response strategy for malaria elimination: Is case reporting, investigation and foci response happening according to plan?
Source: Infect Dis Poverty. 2015 Dec 10;4:55. doi: 10.1186/s40249-015-0089-2 (PMC4674909; doi:10.1186/s40249-015-0089-2)
Supplement: Additional file 1: — Multilingual abstracts in the six official working languages of the United Nations. (PDF 278 kb) [file 40249_2015_89_MOESM1_ESM.pdf]

Translation of the abstract into the six official working languages of the United Nations

استراتيجية 7-3-1 الصينية للمراقبة و الاستجابة من أجل القضاء على الملاريا: هل التبليغ عن الحالة، التحقيق و التصدّي للبؤر يعمل وفقا للخطّة ؟

Shui-Sen Zhou, Shao-Sen Zhang, Li Zhang, Aafje E. C. Rietveld, Andrew R. Ramsay, Rony Zachariah, Karen Bissell, Rafael Van den Bergh, Zhi-Gui Xia, Xiao-Nong Zhou, Richard E. Cibulskis

الملخص

الخلفية: استُهلّت استراتيجية 7-3-1 الصينية و تمّ تبنّيها في أقاليم مختلفة (مناطق جغرافية) للتبليغ عن حالات الملاريا في غضون يوم واحد، ثمّ تأكيدها و فتح تحقيق حولها في غضون ثلاثة أيّام، و أخيرا اجراء الاستجابة الملائمة الخاصّة بالصّحة العامة لتجنّب انتقال العدوى في غضون سبعة أيّام. يُعتبر تقييم مستوى الامتثال لاستراتيجية 7-3-1 على مستوى الإقليم بمثابة الخطوة الأولى لتحديد ما إذا كانت استراتيجية المراقبة و الاستجابة تشتغل وفقا للخطّة أو لا. قيّمت هذه الدراسة إمكانية استمرارية الأهداف المحددة زمنيا لاستراتيجية 7-3-1 على مدى الزمن. تعتبر مثل هذه المعلومات ذات فائدة كبيرة لتحسين تطبيق استراتيجية 7-3-1 في الصّين.

الطُرُق: شملت هذه الدراسة المستعرضة بيانات برمجية من جميع أنحاء البلاد للفترة الممتدّة من 1 كانون الثاني يناير 2013 إلى غاية 30 يونيو 2014. تمّ استخراج متغيّرات البيانات من المنظومة الوطنية الخاصّة بالمعلومات حول الملاريا، كما اشتملت على معلومات اجتماعية و سكّانية، إلى جانل نوع الإقليم، تاريخ التشخيص، تاريخ الإبلاغ، تاريخ التحقيق في الحالة، تصنيف الحالة (حالة موجودة لدى السكان الأصليين، أو حالة مستوردة، أو حالة غير معروفة)، التحقيق المفصّل، تاريخ الكشف عن الحالة التفاعلية (RACD)، و تاريخ الرش الثمالي داخل المباني (IRS). تمّ استعمال إحصاءات موجزة و نِسب و تمّ تقييم المقارنات بين المجموعات باستعمال "اختبار خي مربّع". تمّ ضبط مستوى الدلالة على $P\text{-value} \leq 0.05$.

النتائج: من مجمل 5.688 حالة ملاريا من 731 إقليم، كانت هنالك 55 حالة (1%) موجودة لدى السكّان الأصليّين (فقط في الأقاليم ذات الصنف 1 و الصنف 2) و 5.633 (99%) حالة مستوردة من جميع أصناف الأقاليم. لم يكن هنالك أيّ تأخير في التبليغ عن حالات الملاريا حسب صنف الإقليم. فيما يخص التحقيق في الحالات، تمّ فتح تحقيق في 97.5% من الحالات في غضون ثلاثة أيّام مع نسبة تأجيل بلغت (1.5%) في الصّنف 2 من الأقاليم، و هو ما يجعلها أقلّ بكثير من أقاليم الصنف 1 (4.1%). فيما يخصّ البؤر النشطة، تمت معالجة ما نسبته 96.4% عبر الكشف عن الحالة التفاعلية و/ أو الرش الثمالي داخل المباني.

الخلاصات: كان أداء استراتيجية 7-3-1 مشجّعا مع وجود بعض الصعوبات التي و إن تمّت معالجتها فستحسّن التطبيق بشكل أفضل.

Translated from English version into Arabic by Mokhtar Oussama Alliouche, through

TRANSLATORS
WITHOUT BORDERS

中国消除疟疾 1-3-7 监测与响应策略评估：疟疾病例报告、个案调查及疫点处置都能及时有效开展吗？

周水森, 张少森, 张丽, Aafje E. C. Rietveld, Andrew R. Ramsay, Rony Zachariah, Karen Bissell, Rafael Van den Bergh, 夏志贵, 周晓农, Richard E. Cibulskis

摘要

引言：中国的 1-3-7 消除疟疾病例监测与响应策略是指疟疾病例在诊断后 24 小时（1 天）内报告，3 日内完成病例核实与流行病学个案调查，以及 7 日内完成活动性疫点处置。该策略已成为中国消除疟疾工作的核心措施并在全国不同类型疟疾流行区广泛应用。1-3-7 策略核心指标在县级的执行情况评价是评估该策略在全国执行效果的第一步。本研究通过以县为单位定量评价 1-3-7 策略各项指标完成情况及时效性，为完

善中国 1-3-7 策略的实施提供科学依据。

方法：依据国家疫情信息报告系统横断面调查获得全国 2013 年 1 月 1 日至 2014 年 6 月 30 日的病例监测数据，包括人口学、流行区域分类、疟疾病例报告与分类、个案调查、疫点调查与处置等核心信息。采用描述性统计和卡方分析方法，评价该策略核心指标在不同疟疾流行县的实施效果与时效性，差异统计显著性水平（*P*）设为 0.05。

结果：2013 年 1 月 1 日至 2014 年 6 月 30 日间，全国共有 731 个县报告疟疾病例 5688 例，其中输入病例 5633 例（99%），仅一类县和二类县报告本地感染病例 55 例（1%）。所有病例均在 24 小时内报告，97.5% 的病例个案调查在 3 日内完成。疟疾病例个案调查在不同疟疾流行县均有较小比例延迟，其中二类县延迟比例显著小于一类县（*P*<0.05）。7 日疫点处置完成率为 96.4%。

结论：中国消除疟疾 1-3-7 策略执行情况总体良好，但不同地区仍存在少许困难和挑战需要进一步完善。

Translated from English version into Chinese by Zhang Shao-sen

La stratégie de surveillance et de réponse «1-3-7» chinoise pour l'élimination du paludisme: la notification des cas, leur investigation et le traitement des foyers ont-ils lieu conformément au plan?

Shui-Sen Zhou, Shao-Sen Zhang, Li Zhang, Aafje E. C. Rietveld, Andrew R. Ramsay, Rony Zachariah, Karen Bissell, Rafael Van den Bergh, Zhi-Gui Xia, Xiao-Nong Zhou, Richard E. Cibulskis

Résumé

Contexte: La stratégie «1-3-7» de la Chine a été mise en place et largement adaptée dans différents types de circonscriptions (régions géographiques) pour la notification des cas de paludisme le jour même, leur confirmation et leur examen dans les trois jours et une réponse de santé publique adéquate visant à stopper la transmission dans les sept jours. L'évaluation de l'observance de la stratégie 1-3-7 au niveau des circonscriptions est une première étape pour vérifier si la stratégie de surveillance et de réponse se déroule conformément au plan. Notre étude avait pour but de vérifier si les délais fixés dans la stratégie « 1-3-7 » étaient respectés dans la durée. Ces informations seraient utiles pour améliorer l'application de la stratégie « 1-3-7 » en Chine.

Méthodes: Cette étude transversale portait sur les données programmatiques nationales sur la période courant du 1er janvier 2013 au 30 juin 2014. Les variables, extraites du système national d'information sur le paludisme, comprenaient les informations sociodémographiques, le type de circonscription, la date du diagnostic, la date de notification, la date de l'examen du cas, la classification du cas (indigène, importé ou inconnu), l'examen du foyer, la date de la détection de cas réactive (DCR) et la date de pulvérisation d'insecticide rémanent à l'intérieur des habitations (IRS). Des statistiques résumées et proportions ont été utilisées et des comparaisons entre groupes ont été évaluées à l'aide du test de chi-deux. Le niveau de significativité était fixé à une valeur de *P* de ≤0,05.

Résultats: Sur le total de 5688 cas de paludisme dans 731 circonscriptions, 55 (1 %) étaient indigènes (uniquement dans les circonscriptions de Type 1 et de Type 2) et 5633 (99 %) étaient des cas importés en provenance de tous les types de pays. Il n'y a pas eu de retard dans la notification des cas de paludisme en fonction du type de pays. En ce qui concerne l'investigation des cas, 97,5 % des cas ont été examinés dans les 3 jours, avec une proportion de retards (1,5 %) significativement plus faible dans les circonscriptions de Type 2 que dans celles de Type 1 (4,1 %). Un traitement par DCR et/ou par IRS a été réalisé dans 96,4 % des foyers actifs.

Conclusions: Les résultats de la stratégie «1-3-7» sont encourageants mais nous avons identifié quelques difficultés qu'il faudra résoudre afin d'améliorer encore son déploiement.

Translated from English version into French by Suzanne Assenat, through

Стратегия 1-3-7 наблюдения и ответных мер для ликвидации малярии в Китае: насколько отчётность, изучение заболеваний и подавление их очагов проводится в соответствии с планом

Шуй-Сен Чжоу (Shui-Sen Zhou), Шао-Сен Чжан (Shao-Sen Zhang), Ли Чжан (Li Zhang), Аафдже Е. С. Ритфельд (Aafje E. C. Rietveld), Эндрю Р. Рэмси (Andrew R. Ramsay), Рони Захария (Rony Zachariah), Карен Бисселл (Karen Bissell), Рафаэль ван ден Берг (Rafael Van den Bergh), Чжи-Гуй Ся (Zhi-Gui Xia), Сяо-Нун Чжоу (Xiao-Nong Zhou), Ричард Е. Цибульскис (Richard E. Cibulskis)

Аннотация

Краткое описание: Разработанная в Китае стратегия 1-3-7 применяется и широко распространилась в разных округах (географических регионах) страны. В соответствии с этой стратегией о случаях заболевания малярией следует сообщать в течение одного дня, подтверждение и изучение заболеваний должно происходить в течение трех дней, и надлежащие здравоохранительные меры по предотвращению распространения заболеваний должны быть приняты в течение семи дней. Оценка соблюдения требований стратегии 1-3-7 на уровне округов является первым шагом для определения того, насколько наблюдение и ответные меры осуществляются согласно плану. Данное исследование оценивает то, как сроки стратегии 1-3-7 соблюдаются на протяжении продолжительного периода времени. Подобные сведения могут быть полезными для улучшения внедрения стратегии 1-3-7 в Китае.

Методы: В ходе исследования, охватившего разные типы округов, были обработаны программные данные по всей стране за период с 1 января 2013 года по 30 июня 2014 года. Эти данные были получены из национальной информационной системы о малярии и включали социально-демографическую информацию, тип округа, дату диагностирования, дату сообщения о заболевании, дату его изучения, классификацию случая заболевания (местное, завезенное или неизвестно), ключевые положения исследования, дату реактивного выявления заболевания (RACD) и дату распыления инсектицидов остаточного действия внутри помещений (IRS). Были использованы суммарные статистические данные и пропорции. Сравнения между группами были оценены с использованием критерия хи-квадрат при уровне значимости P-value ≤ 0,05.

Результаты: Из общего числа 5688 заболеваний малярией в 731 округах 55 заболеваний (1%) были местными случаями заражения (только в округах 1-го и 2-го типов) и 5633 заболеваний (99%) завезенными случаями заражения для всех типов округов. Задержек с сообщением о заболеваниях не было во всех типах округов. В отношении изучения заболеваний 97,5% случаев расследовались в течение 3 дней, при этом процентное отношение задержек в округах 2-го типа составило 1,5%, что значительно ниже 4,1% задержек в округах 1-го типа. Показатели подавления очагов заражения RACD и/или IRS составили 96,4%.

Заключение: Результаты применения стратегии 1-3-7 являются обнадёживающими, однако были выявлены отдельные трудности, устранение которых приведет к дальнейшему улучшению внедрения стратегии.

Translated from English version into Russian by Natalia Potashnik, through

TRANSLATORS
WITHOUT BORDERS

La estrategia 1-3-7 de monitoreo y respuesta para la eliminación de la malaria en China ¿Se está siguiendo el plan en el reporte de casos, la investigación y la respuesta a focos?

Shui-Sen Zhou, Shao-Sen Zhang, Li Zhang, Aafje E. C. Rietveld, Andrew R. Ramsay, Rony Zachariah, Karen Bissell, Rafael Van den Bergh, Zhi-Gui Xia, Xiao-Nong Zhou, Richard E. Cibulskis

Resumen

Antecedentes: La estrategia 1-3-7 de China se inició y adoptó ampliamente en distintos tipos de condados (regiones geográficas) para el reporte de los casos de malaria dentro de las 24 horas, su confirmación e investigación dentro de los tres días y la respuesta de salud pública adecuada para prevenir una mayor transmisión dentro de los siete días. Un primer paso hacia la determinación sobre si la estrategia de monitoreo y respuesta está funcionando de acuerdo al plan, es la evaluación del nivel de cumplimiento de la estrategia 1-3-7 a nivel de condado. Este estudio evaluó si los objetivos de tiempo limitado de la estrategia 1-3-7 se mantenían en el tiempo. Dicha información será útil para mejorar la implementación de la estrategia 1-3-7 en China.

Métodos: El estudio transversal incluyó datos programáticos de todo el país para el período del 1ro de enero de 2013 al 30 de junio de 2014. Las variables se extrajeron del sistema nacional de información de la malaria que incluía información socio-demográfica, tipo de condado, fecha del diagnóstico, fecha del reporte, fecha de la investigación del caso, clasificación del caso (local, o importado, o desconocido), investigación del foco, fecha de la detección reactiva del caso y fecha de rociado residual de interiores. Se utilizaron resúmenes de estadísticas y proporciones y se evaluaron las comparaciones entre los grupos mediante el uso de la prueba de chi-cuadrado. El nivel de significancia se estableció en un Valor $P \leq 0.05$.

Resultados: De un total de 5.688 casos de malaria de 731 condados, hubo 55 (1%) de casos locales (solo en los condados Tipo 1 y Tipo 2) y 5.633 (99%) de casos importados de todo tipo de condado. No hubo demoras en el reporte de los casos de malaria por tipo de condado. En términos de la investigación de casos, 97,5% de los casos se investigaron dentro de los 3 días con una proporción de demoras (1,5%) en los condados tipo 2, siendo mucho menor en los condados de tipo 1 (4,1%). En cuanto a los focos activos, 96,4% fueron tratados con detección reactiva del caso y/o rociado residual de insecticida.

Conclusiones: El desempeño de la estrategia 1-3-7 fue alentador pero identificó algunos desafíos, que si se abordan, pueden mejorar aún más su implementación.

Translated from English version into Spanish by Maria Alejandra Aguada, through

TRANSLATORS
WITHOUT BORDERS
